# Polymer Versus Cementitious Matrix Composites for Retrofitting Reinforced Concrete Columns—A State-of-the-Art Review

**DOI:** 10.3390/polym17212865

**Published:** 2025-10-27

**Authors:** Hussein Elsanadedy, Aref Abadel, Husain Abbas, Tarek Almusallam, Yousef Al-Salloum

**Affiliations:** Chair of Research and Studies in Strengthening and Rehabilitation of Structures, Department of Civil Engineering, College of Engineering, King Saud University, P.O. Box 800, Riyadh 11421, Saudi Arabia; aabadel@ksu.edu.sa (A.A.); habbas@ksu.edu.sa (H.A.); musallam@ksu.edu.sa (T.A.); ysalloum@ksu.edu.sa (Y.A.-S.)

**Keywords:** polymer matrix composites, FRP, cementitious matrix composites, FRCM, retrofitting, RC columns

## Abstract

Fiber-reinforced polymer (FRP) composites have become a popular solution for upgrading reinforced concrete (RC) structures due to their corrosion resistance, high strength-to-weight ratio, and speed of implementation. However, their organic resin binder has issues, including temperature sensitivity, poor performance in moist conditions, a high cost, and potential health risks. Additionally, reversing FRP repair can be difficult and may damage the original structure, posing a significant reversibility issue. A promising alternative to FRP is the fiber-reinforced cementitious matrix (FRCM), which replaces the organic resin with an inorganic cementitious mortar. This new class of composite uses a breathable textile instead of the tightly packed fibers in FRP. The present article provides a comprehensive assessment of the two composites (FRP and FRCM) used for the retrofitting of RC compression members, with the purpose of identifying existing knowledge gaps and outlining future research objectives. The materials used in different strengthening approaches using both FRP and FRCM have been identified, and their stress–strain characteristics under tensile load have been outlined. The study also explores techniques of implementation using the two materials. This study presents available studies comparing the utilization of FRCM composites with FRP for the axial retrofitting of RC compression members in both ambient and high-temperature conditions.

## 1. Introduction

The strengthening of existing structures, especially concrete, has become very common over the past few years owing to a variety of reasons, including (1) the aging of construction materials, thereby causing damage to structural elements; (2) modifications to the structure, such as the removal of columns or walls or making new openings in slabs; (3) increases or changes in loading as a result of increased live load, and new installations such as heavy machinery; (4) retrofitting for seismic loading to fulfill current seismic code requirements; and (5) design or construction errors, such as structural elements deficient in reinforcement or the design dimensions being deficient.

**Figure 1:**
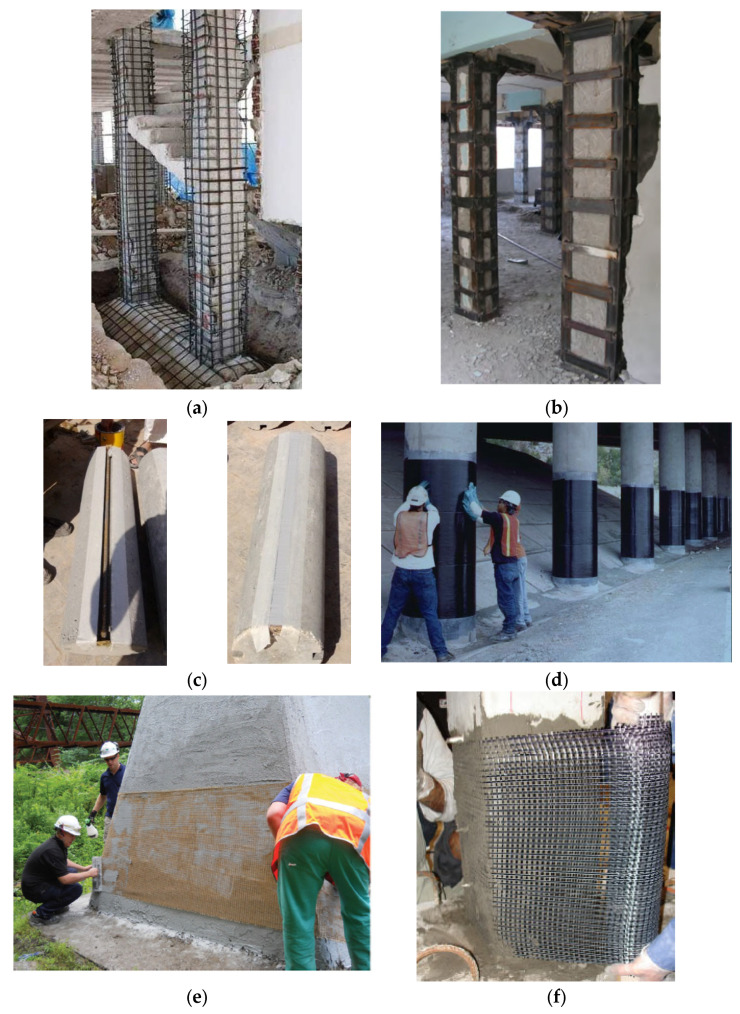
Examples of methods used to strengthen RC columns: (**a**) section enlargement [7]; (**b**) Steel jacketing [8]; (**c**) NSM strengthening [9]; (**d**) FRP jacketing [10]; and (**e**) FRCM jacketing [5,6].

Commonly used upgrading techniques for reinforced concrete (RC) columns include the jacketing of columns using steel, the enlargement of column sections, fiber-reinforced polymer (FRP) sheets being bonded externally, or steel or glass FRP (GFRP) rebars being used as near surface mounted (NSM) bars [1,2,3,4]. Some of these techniques, illustrated in Figure 1 [5,6,7,8,9,10], are briefly discussed below.

The enlargement of a section is a straightforward strategy for upgrading columns and is also simple in its implementation (Figure 1a). It involves placing new concrete and steel rebars or other reinforcement in a formwork around an existing section and increasing the section size of a column, thereby increasing its load carrying capacity and stiffness. In this type of strengthening, it is important to ensure that the new section performs monolithically with the old section [11]. This method has been found to be simple in design as well as efficient and cost-effective. However, the reinforced concrete section needs to be enlarged significantly to increase the column capacity in an efficient manner, which, in turn, imposes additional loads and might infringe on clearance limits. Furthermore, this method involves steps such as formwork construction and the placing of reinforcement, which are labor-intensive [1].

Steel jacketing is another form of strengthening commonly used for RC columns. It involves encasing columns using a steel jacket in the form of steel plates and angles anchored to column concrete or steel shells in combination with non-shrink grouts to fill the gaps, thereby providing passive confinement to the concrete core (Figure 1b). However, due to the high tensile modulus for steel, the jacket attracts a major portion of axial force, which may lead to the premature buckling of the steel shell.

NSM rebars can be mounted after making grooves in the concrete cover and by using cementitious mortar or adhesive binder as fillers to bond steel or FRP rebars to the old concrete (see Figure 1c). NSM reinforcement first began in Europe and was mainly performed using steel rebars; however, with the recent advancements in composite materials such as FRPs and epoxy/resins, this method is now being used along with the composite materials, and both the research community as well as practicing engineers have often used this method as a tool to strengthen RC columns [12].

FRP materials have become more popular as strengthening materials for RC structural members since the early 1980s. This popularity could be attributed to several benefits that these materials offer over conventional materials, like resistance to corrosion, minimal sectional enlargements, a higher strength compared to their weight, and, more importantly, the ease and rapidity of their application [13]. The process of using externally bonded FRP composites in the upgrading of RC columns involves wrapping the columns using the composites, which provides passive confinement, thereby increasing their load capacity and ductility. The orientation of the FRP fibers is orthogonal to the longitudinal axis of the column, and the fibers are glued to the column surface using epoxy resin. Examples of FRP jackets used for strengthening of columns are presented in Figure 1d.

With all its advantages, the FRP strengthening method does have a few disadvantages, and most of them can be attributed to the use of resins for binding the FRP sheets to the RC [5,14,15]. Some of the drawbacks include the steep cost of epoxies, poor performance of polymer-based adhesives at higher temperatures (in the range beyond the glass transition point), FRP de-bonding, non-compatible substrate and epoxy materials, vapor permeability problems, and difficulties in applying FRP composites in wet environments. Moreover, it is extremely difficult to assess the damage suffered by concrete elements after a seismic event, when the FRP remains undamaged.

To overcome the above disadvantages of the FRP strengthening method, the most likely solution is to swap organic resins with cementitious mortars, which would lead to the use of fabric-reinforced cementitious matrix (FRCM) instead of FRP [16]. Unlike epoxy resins, mortars, however, do not wet individual fibers, and, due to their granularity, complete impregnation of fiber sheets is not easily achieved. For this reason, in order to achieve an enhanced fiber–matrix interaction, fiber sheets that are continuous are replaced by fabric textiles with meshes. These fabrics consist of knitted, woven, or unwoven fiber rovings, oriented along two or more orthogonal directions. The mechanical characterization of the fabric can be varied and the amount of cementitious mortar that penetrates through the fiber mesh can be controlled by varying the distance between the fiber rovings in the two orthogonal directions. The combined action in the fabric and mortar is developed through the interlock, which depends on the degree of penetration of the mortar matrix. In order to circumvent the disadvantages associated with FRPs, new materials have recently been introduced into the construction industry [6]. These materials are referred to by various designations, including textile-reinforced mortar (TRM), inorganic matrix composites, FRCM systems, fabric RC systems, and mineral-based composites. Figure 1e depicts examples of the retrofitting of RC compression members utilizing FRCM composites.

FRCM systems are particularly preferred in applications that demand enhanced temperature resistance, vapor permeability under high humidity conditions, and reversibility, such as in heritage conservation or fire-exposed structures, since they eliminate the drawbacks of epoxy-bonded FRP systems, including a reduced performance at elevated temperatures and moisture sensitivity during application [6,17].

This article aims to provide a state-of-the-art review of the available research for comparisons between organic-epoxy-resin-based FRPs and inorganic matrices used in the retrofitting of RC columns. It also identifies the existing gaps in the present scientific understanding of this topic and outlines different trends that future research on this topic can pursue. Section 2 of this paper includes the characterization of constituent materials as well as their stress–strain behavior. Installation methods for both organic and inorganic composite systems are also described concisely in this section. The available research and a discussion on their main findings on the use of inorganic versus organic matrix composites in confinement of RC columns under the effect of elevated temperatures have been presented in Section 3 of this paper. Section 4 presents a review of the existing code provisions and design guidelines for FRP and FRCM systems for the retrofitting of RC columns. Finally, the conclusions and suggestions for the extension of this research are highlighted in Section 5.

## 2. Characterization of FRP and FRCM Materials

The stress–strain behavior in tension and the material properties for organic and inorganic composites have been characterized in this section. The methods used for the installation of the composite system on RC columns have also been discussed. The material characterization for FRCM composites has been provided in detail in this section, as they are comparatively new materials. However, the FRP composites have been in use for a while now, and their material properties are well-documented in the literature [18]. For this reason, FRP’s material properties have been briefly outlined in this section.

### 2.1. FRP Composites

**Figure 2:**
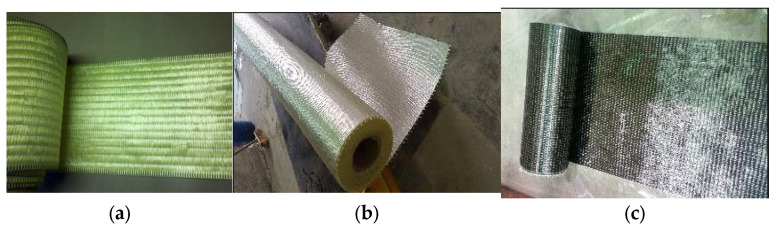
Types of sheet fibers utilized in FRP system: (**a**) aramid [30]; (**b**) glass (photo taken by H.M. Elsanadedy); and (**c**) carbon (photo taken by H.M. Elsanadedy).

**Figure 3:**
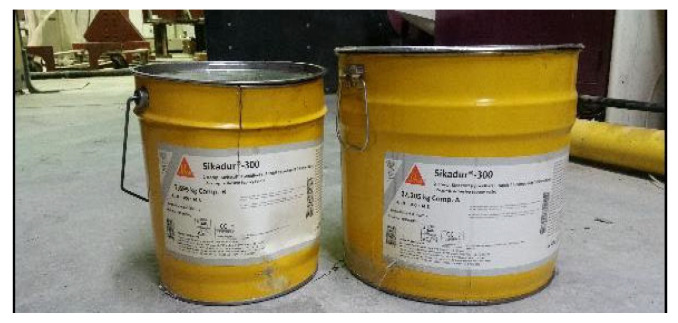
Example of organic matrix used in FRP composites (SIKADUR 300 epoxy resin) (photo taken by H.M. Elsanadedy).

**Figure 4:**
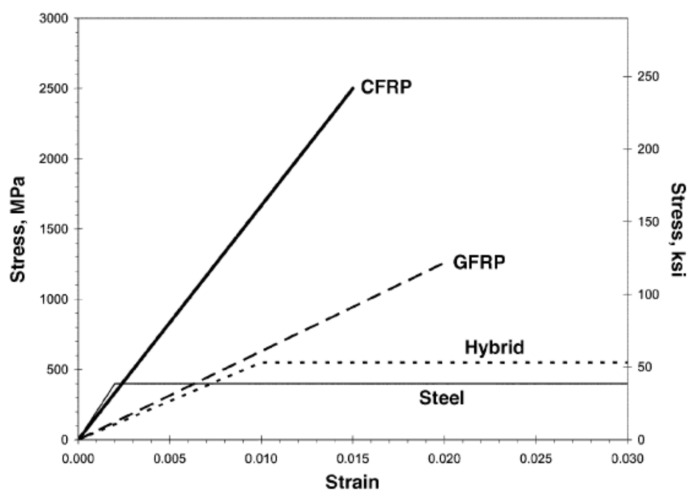
Typical stress–strain curves for FRP composites [18].

FRP materials date back to the 1970s, and research over a long period has resulted in their widespread use in building construction, for improving the capacity of structural elements [19,20,21,22,23,24]. Apart from their employment as a structural material in building construction, FRPs have also been employed in other areas [18,25,26]. FRP combines high-tensile-strength fibers that are strong and lightweight. Fibers in FRP are made from different materials, but the ones that have gained the most attention include glass, carbon, and aramid. The material characteristics of these fibers can have many variations, and that depends on the manufacturer as well as the type of fiber [18,23,26]. These fibers are shown in Figure 2. Table 1 enlists an interesting comparison between the various fiber types commonly employed in FRP systems. The binder in the FRP, which is the other component, is used to impregnate the fibers and is a thermosetting organic polymer such as epoxy, vinyl-ester, polyester, phenolics, or polyurethane (see Figure 3). The combination of the fibers and the polymer makes FRP an efficient system with stress transfer through the interfacial bond. FRP’s mechanical properties, e.g., strength in tension and elastic modulus, are determined by carrying out tensile tests on FRP coupons as per the ASTM D3039/3039M [27] standard. The typical stress–strain behavior for both the FRP material and steel is depicted in Figure 4. The figure indicates that the FRP material behaves in a linear elastic manner, unlike the steel, where the curve shows an elasto-plastic behavior. Further information regarding the material characterization of FRP composites is available in other sources [18,28,29].

**Figure 5:**
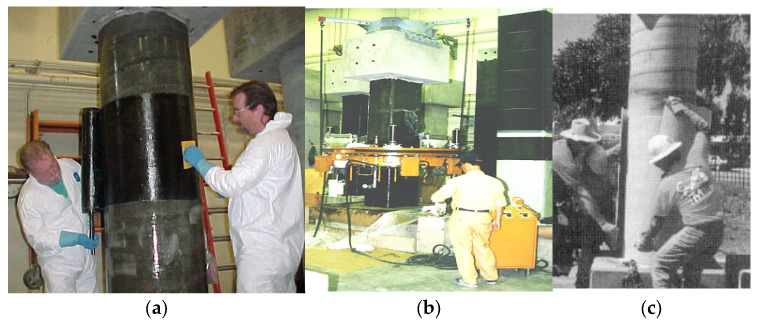
Installation techniques for FRP systems include [29]: (**a**) hand layup; (**b**) machine wrapping; and (**c**) pre-cured systems.

The installation of FRP systems onto a concrete surface should usually follow the recommendations given by the manufacturer. The concrete or other installation surfaces should be dry before the FRP system is applied, as water can inhibit the penetration of resin, thereby reducing the mechanical interlock. The assessment of the moisture content shall follow the guidelines of ACI 503.4 [32]. Surface preparation before the application of FRP involves sandblasting, cleaning, and brushing, and is a necessity in order to ensure a proper bond. The methods of FRP installation can be broadly classified into three types: machine wraps, hand layup, and pre-cured systems. Hand layup (also called wet layup) involves placing uncured fabric on the column by hand. The fabric, which is in wide rolls, is cut to length for ease of handling. The wide fabrics are commonly infiltrated with liquid resin by dipping the cut length in a bath. The epoxy-saturated fabric is laid onto the prepared concrete surface and spread by hand to smooth the fabric and release any trapped air. This technique is depicted in Figure 5a for the FRP wrapping of RC columns. The automated wrapping of RC columns (Figure 5b) is primarily accomplished using wrapping machines wherein the fibers are directly wrapped onto the column from a spool. When wrapping is performed using a machine, both pre-impregnation and on-line impregnation, where the fibers are passed through an epoxy bath just before wrapping, can be employed. Once wrapping is accomplished, prepreg systems need curing at pre-determined temperatures by keeping a heat source close to the RC columns, as per the recommendations of the manufacturer. Pre-cured systems consist of open-grid forms, or shells and strips that are installed using an adhesive (Figure 5c). Once the surface is prepared to receive the FRP system, the epoxy is uniformly applied to the surface, and the pre-cured shells or sheets are placed on or within the wet adhesive as per the manufacturer’s recommendations. All entrapped air across the laminar interface of layers should be removed before the adhesive sets.

### 2.2. FRCM Composites

**Figure 6:**
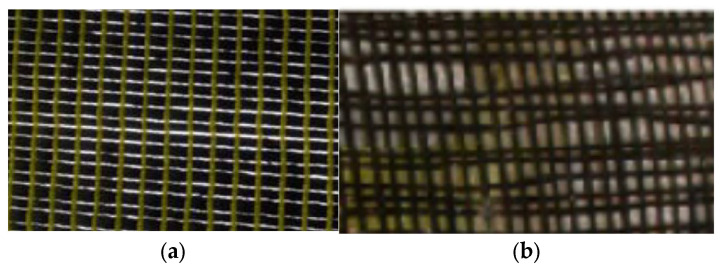
Various types of fiber reinforcement (photos taken by H.M. Elsanadedy): (**a**) carbon fiber sheet; and (**b**) carbon fabric.

FRCM garnered the interest of the research community in Europe as a promising substitute to the FRP system for strengthening RC compression members. FRCM research started in the 1980s, and continued through the 1990s; however, focused efforts were initiated only from 2002 onwards [6,33,34,35]. FRCM is applied to structures in a manner similar to FRP; however, it overcomes the major drawbacks of FRP systems, as an inorganic cementitious matrix replaces the polymer matrix as the binder. In early research attempts, it was found that the cementitious binder could not impregnate the continuous fiber sheets as effectively as the epoxy resin [15,20,36,37,38,39,40]. For this reason, open-mesh textiles of fabrics were introduced as a replacement for sheets (Figure 6), which led to a much better interlock, thereby enhancing the bond between the composite and cementitious binder [6].

#### 2.2.1. Fabrics and Textiles

**Figure 7:**
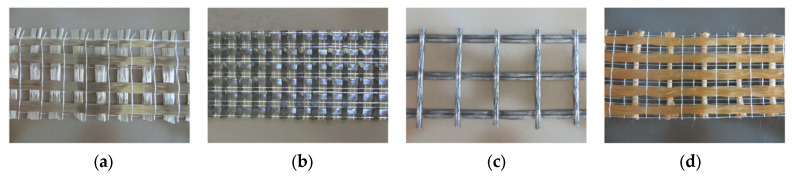
Textile fabrics employed in FRCM composites [3]: (**a**) basalt; (**b**) carbon; (**c**) glass; and (**d**) PBO.

**Figure 8:**
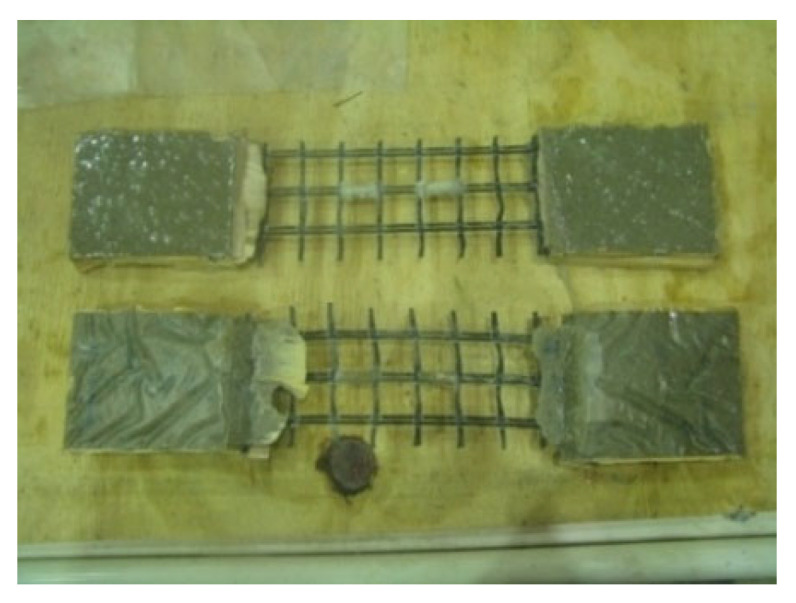
Fabric coupons [39].

FRCM is fundamentally similar to FRP and is occasionally referred to as its “twin brother” [6], differing primarily in two key aspects: the resins and sheets are replaced by mortar and textiles, respectively. As described earlier, the textile or fabric, which is a continuous dry woven mesh in two mutually perpendicular directions, is bonded to the concrete surface using a cementitious mortar. In order to improve durability or manufacturing, a small amount of epoxy resin is sometimes used on some strands, but the epoxy does not impregnate the fibers. For this reason, the term dry fabric is still valid [6,19,20,22,39,41,42]. Typical materials used as fabrics or textiles in FRCM include aramid, carbon, glass, basalt, and polyphenylene benzobisoxazole (PBO), which are all high-performance fabrics known for their excellent mechanical properties, corrosion resistance, improved fatigue performance, and reduced manufacturing costs [43]. These fabrics are available in a variety of patterns as well, and are sourced based on specific applications. Glass fibers have adverse effects when exposed to the alkaline environments of the cement matrix; therefore, only alkaline-resistant designated glass should be used in FRCM [44]. Figure 7 shows the four fabric types commonly employed in research studies. The properties of the bare dry fabric are usually provided by the manufacturer; however, experimental testing is required to come up with the actual properties of the material. Uniaxial tension tests are typically conducted on test coupons of the bare textiles, as depicted in Figure 8 [39].

#### 2.2.2. Mortars

**Figure 9:**
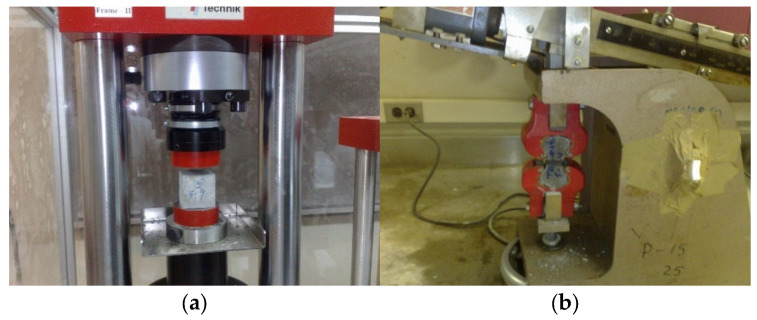
Standard methods of testing mortar samples [49]: (**a**) cube in compression; and (**b**) briquette specimens in tension.

Mortar selection is essential to the performance of FRCM, as the binder performance is not necessarily based on its tensile or compressive strength and elastic modulus, but depends on the quality of the bond between the mortar and substrate, and fabric and mortar. Mortar can be modified to suit the fabric being used by changing the properties of its constituent materials, e.g., using high fineness cement, additives, micro aggregates, adhesion promoters, polycarboxylic, and fly ash [6,19]. The mechanical properties of the mortar used in FRCM composites are usually determined by experimental testing. The compressive strength of mortars can be determined by carrying out compression tests on standard 50 mm cubes as per the relevant test standard [45], as seen in Figure 9a. This figure shows a standard tensile test for the mortar in which briquette samples are prepared and tested in compliance with Ref. [46]. The mechanical properties for some commercially produced polymer-modified cementitious mortars are listed in Table 2. In FRCM systems, the mortar is responsible for transferring the loads to the textile fabric, and, for this reason, it should fulfill specific general requirements. The mortar used in FRCM systems should be shrink-resistant, highly workable, suitable for trowel application, and possess a high viscosity so that it can be applied on vertical as well as overhead surfaces. It should have an appropriate shear strength to avoid premature debonding and maintain its workability over time with a low rate of slump loss [47]. Mortars are either of the cementitious or polymer-modified cementitious type [39], and, in both, Portland cement is used as a base material. Polymer-modified cementitious mortars have a dry polymer dosage of less than 5% by weight of cement, which serves to improve matrix properties such as bond, workability, setting time, and their mechanical performance [6,48].

#### 2.2.3. Composite Properties

A considerable amount of literature is available on the response of FRCM composites to tensile loading. A document that enlists the acceptance criteria for the tensile testing of FRCM systems has been published by the ICC Evaluation Service [41] and includes details on casting single or multiple layers of FRCM specimens in wooden forms, as well as dimensioning and gripping techniques. The test gripping method and the performance of the bare textile and mortar have an important role in the tensile response of the FRCM composite.

**Figure 10:**
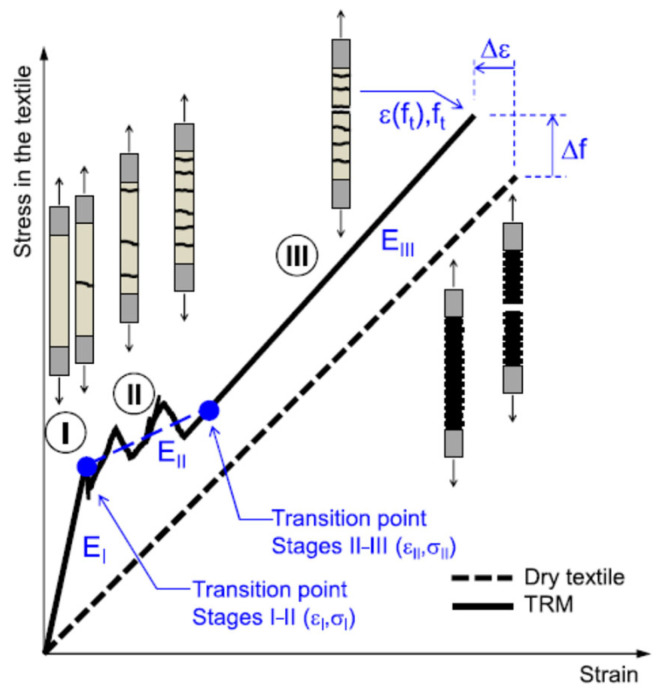
FRCM tensile response stages [52].

The tensile stress–strain response of FRCM is non-linear with three stages, as depicted in Figure 10. In the initial Stage I, the mortar remains uncracked and contributes to both the strength and material stiffness. Considering an equivalent homogenized section and considering a perfect bond between the textile and matrix, the modulus of elasticity of FRCM (E_I_) can be estimated in this stage. Stage II begins with the appearance of the first crack in the mortar matrix. The stress and strain values (σ_I_, ε_I_) help identify the transition point between Stages I and II, with higher σ_I_ values indicating a higher tensile strength and better mortar-to-fabric bond. The calculation of stresses, based on the size of the dry textile section, indicates that altering the textile density proportionately affects both stress and stiffness in Stage I. Therefore, the behavior of FRCM composites with bigger fiber meshes (e.g., PBO) and high-performance mortars (e.g., fiber-reinforced cement composites with polymer additives) is mainly governed by their behavior in Stage I [50,51].

With the onset of Stage II, cracks grow progressively, and the formation of cracks typically causes load drops, resulting in an uneven stress–strain curve. The stiffness in this region, E_II_, is generally determined through linear interpolation over an appropriate data range. For this reason, the gauge length (the length in which displacement/strain is monitored) should be sufficiently large to include a large number of cracks. The crack patterns govern the properties of the FRCM composite, and E_II_ should be evaluated based on the response curve and the cracks that actually develop. However, when only a single crack appears, E_II_ may not be well-defined [52]. The E_II_ calculated in this stage is much lower compared to E_I_, as the cracking of the matrix results in large strain increments with smaller corresponding stress increases. Once the crack pattern fully develops, a significantly stiffer response is noted, with the increase in strains being directly proportional to the increase in stresses, indicating the start of Stage III. The transition point is identified by the intersection of the linear regression line from Stage II with the curve of Stage III. No further new cracks are developed in Stage III; however, the existing cracks widen as the displacement increases. The gradient of the response curve in this stage is designated as E_III_. The end of Stage III culminates with the stress peaking at f_t_ and strain at ε_t_. FRCM systems comprising large volumes of stiff textiles (e.g., steel-reinforced) bonded with a mortar-based matrix exhibit behavior primarily governed by Stage III. The initial two stages are nearly indistinguishable in FRCM materials where dense fabrics are integrated with relatively weak or flexible matrices [53]. In Stage III, the strength and stiffness of the composite predominantly depend on the textile; thus, the response approaches that of the bare fabric. In some cases, FRCM exhibits a higher elastic modulus and a lower strain at peak load compared to the dry textile. This could be due to the mortar matrix contributing additional stiffness between subsequent crack development, more uniform stress distribution among filaments, or the localized damage in the grips causing the premature rupture of some of the filaments. Consequently, the strength increase when transitioning from dry fiber to FRCM samples may be due to the under-estimation of the textile tensile strength when tested alone [51]. This disparity of behavior between dry fibers and FRCM composites depends on the interlock between the mortar and fabric, which is greater for dry textiles fabricated from multi-filament materials, e.g., glass, carbon, and basalt, and lower for steel textiles and pre-impregnated or coated fabrics [52]. Typical ranges for the stiffness at each stage, along with the maximum strength and strain values, based on the recent literature, are presented in Table 3 for different types of FRCM composite systems [22,48,53,54].

#### 2.2.4. Installation Procedure

**Figure 11:**
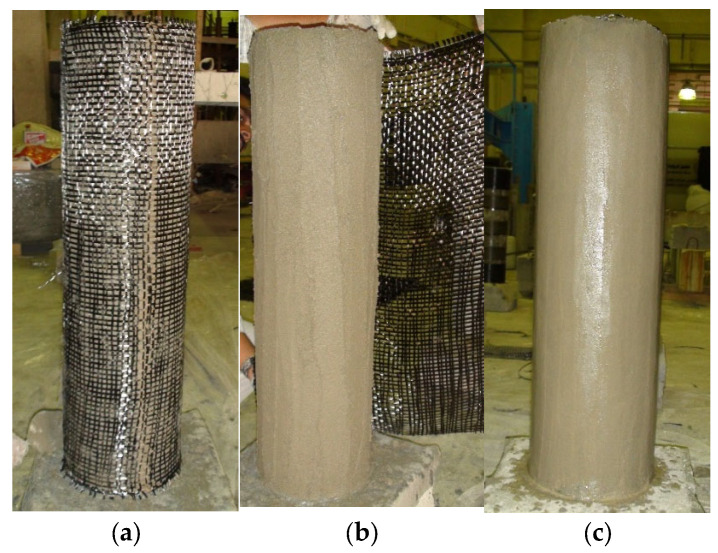
Strengthening of RC column using FRCM composite system [9]: (**a**,**b**) first and second layers of FRCM applied; and (**c**) after applying 3 layers of FRCM.

The installation procedure for FRCM systems is illustrated in Figure 11. Surface preparation is performed before installing the FRCM systems, which may include sandblasting, cleaning, and brushing to ensure the suitability of the substrate for proper bonding. After surface preparation, a 2 mm-thick mortar layer is first applied to the concrete substrate. The fabric is then applied and pushed onto the mortar so that the mortar protrudes out through the openings in the fabric mesh. The textile fabric is then completely covered with the next layer of mortar. This process is repeated for each layer of FRCM. The final finishing mortar layer is then applied and levelled using a trowel. It should be noted that each layer of FRCM should be applied on the previous wet layer to achieve a good bond between successive layers.

## 3. FRP Versus FRCM Composites in Confinement of RC Columns

This section presents a summary of the research conducted on the performance comparison of RC columns wrapped with externally bonded FRP versus FRCM jackets. In addition, discussions of the main findings and outcomes are outlined.

### 3.1. Summary of Conducted Research

Even though several research publications are available on concrete columns confined with FRP composite jackets, only limited studies exist on the response of columns wrapped with FRCM jackets. Studies comparing the two composite systems for the confinement of concrete columns are particularly rare. A few key publications in this regard are summarized below.

Wu and Sun [56] conducted an experimental study comparing the effectiveness of epoxy- and cement-based composite thin sheets for structural strengthening. The experimental campaign consisted of strengthening concrete cylinders using carbon-fiber-reinforced cement (CFRC) and carbon FRP (CFRP) sheets. The results indicated that the CFRC-wrapping of cylinders substantially enhanced their load capacity, with compressive strength values comparable to those of CFRP-strengthened specimens. There was also a considerable improvement in the ductility of the strengthened cylinders, resulting in a less explosive failure compared to CFRP wrapping.

In another experimental study, researchers [36] compared the confinement effectiveness of FRCM and FRP jackets in enhancing the axial load capacity of concrete. To achieve this aim, they tested unreinforced concrete cylinders (150 × 300 mm) and short square columns (250 × 250 × 700 mm), with variables including the type of matrix (epoxy resin versus inorganic mortar), type of inorganic binder (two different types: Mortar I and Mortar II), number of strengthening layers (2, 3, or 4), and bonded versus unbonded jackets. The results indicated the following: (a) FRCM confinement significantly improved both strength and deformability, with the tensile strength of the mortar governing failure modes (fiber rupture or debonding), and strength gains increasing with the number of layers; (b) compared to FRP, FRCM was less effective, with the strength enhancement (with respect to the control) reduced by up to 80% and ultimate strain by up to 50% (with respect to the control), though the performance depended on mortar type and could be improved by modifying its composition; (c) mortar-impregnated textile jackets failed in a more gradual and less explosive manner than resin-based jackets; and (d) FRCM was also effective for square columns, providing strength levels comparable to FRP and only slightly lower ultimate strains.

Another study [57] investigated the comparative effectiveness of FRCM and FRP jackets for confining RC columns by testing 15 stub RC columns under uniaxial compression. The columns were divided into three groups and the specimen sizes were selected to represent approximately 2/3-scale columns. The first group had no internal reinforcement, whereas the second and third groups had 4Ø12 mm longitudinal bars with Ø8 mm ties. The spacing of the ties was varied: 200 mm c/c in the second group (simulating outdated detailing), and 100 mm in the third group (representing the current practice). Each group had five specimens with the following configuration: an unwrapped control, two- and three-layer FRP-wrapped specimens, and four- and six-layer FRCM-wrapped specimens. The specimens strengthened using FRCM jackets had twice the number of layers in the circumferential direction compared to that of FRP, in order to have equivalent confinement levels. The study was aimed to assess the jacket performance in relation to the number of layers (volumetric ratio), internal reinforcement, and stirrup spacing. The results showed that FRCM confinement considerably increased the deformability and strength, with improvements proportional to the volumetric ratio of the FRCM wrap, largely due to the delayed buckling of the longitudinal bars. Although FRCM jackets were marginally less efficient than FRP (by about 10% in strength and deformability), they still provided substantial confinement benefits.

Di Ludovico et al. [58] performed extensive tests and concluded that the basalt-reinforced mortar (BRM) technique could be an alternative for overcoming the drawbacks of epoxy-based FRP laminates. This conclusion was drawn based on the results from testing 23 concrete cylinders of low compressive strengths and confining them with (a) epoxy- or latex-impregnated basalt fibers bonded using cementitious mortar, and (b) GFRP laminate jackets. The study aimed to compare the efficacy of the confinement provided in terms of strength and ultimate strain gains among different confining techniques. The study’s outcome revealed that the BRM confinement technique provided considerable enhancement in both the deformability and strength of concrete compared with unwrapped specimens, and resulted in less brittle failures compared to GFRP-wrapped specimens.

**Figure 12:**
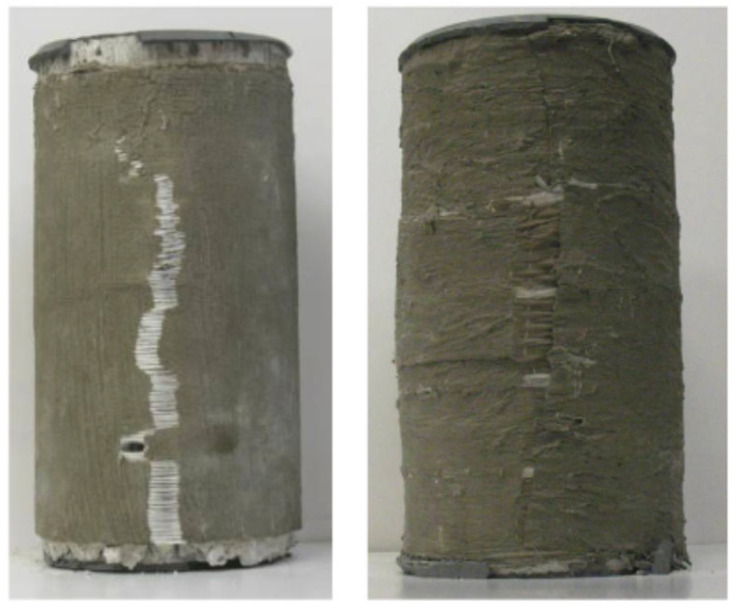
Modes of failure of FRC-confined cylinders tested in Ref. [59].

**Figure 13:**
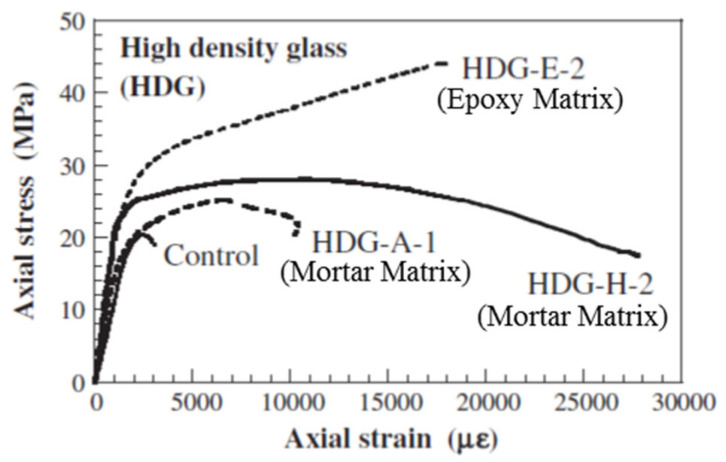
Stress–strain curve of representative cylinders [59].

A study [59] was conducted to explore the development of a bidirectional fiber-reinforced cement (FRC) matrix for improving fire resistance. The initial phase focused on selecting an optimal FRC system from various combinations of fibers and inorganic matrices. The selection was based on constructability, strength, ductility, and compatibility. Constructability factors included workability and ease of application, while compatibility depended on the FRC’s quality, interfacial bond, and ease of fiber impregnation. In the second phase of the study, confined cylinders prepared using the selected FRC system were tested under uniaxial compression. The main parameter studied was the FRC reinforcement ratio, assessed through axial and in-plane deformation measurements as a factor affecting confinement effectiveness. Finally, reversing the FRC application was investigated by breaking the FRC jacket’s bond with concrete by introducing bond-breaking chemicals. The results showed that the selected FRC system substantially increased deformability as well as strength compared to unconfined cylinders. Figure 12 shows the failure modes of FRC-confined cylinders. As seen from the figure, the failure occurred due to compatibility issues in the jacket, caused by the separation between the fiber reinforcement and the inorganic matrix. This failure mechanism results in a stress–strain behavior characterized by continued axial deformation beyond the ultimate stress point, differing from the brittle failure typical of FRP-wrapped specimens (see Figure 13). SEM (scanning electron microscope) images revealed that failures in FRC-wrapped specimens due to the separation of fibers from the inorganic matrix could be because of the poor or incomplete fiber impregnation, which could be improved to achieve a higher efficacy in the use of FRC materials. Semi-empirical models for deformability and compressive strength of concrete wrapped with FRC were also presented in this study.

**Figure 14:**
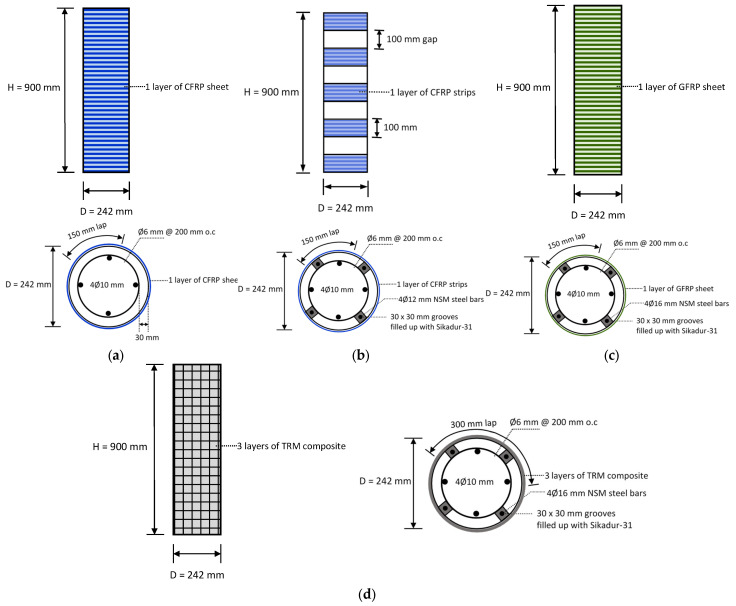
Different column strengthening groups tested by Al-Salloum et al. [9]: (**a**) CFRP; (**b**) CFS/NSM; (**c**) GFRP/NSM; and (**d**) FRCM/NSM.

**Figure 15:**
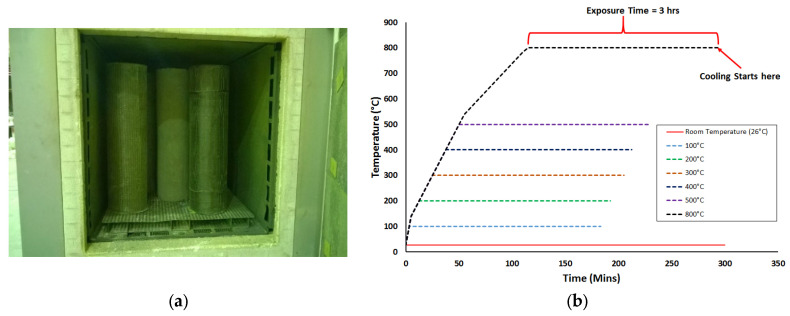
Heating of column specimens tested by Al-Salloum et al. [9]: (**a**) specimens in oven before heating; and (**b**) temperature plots.

**Figure 16:**
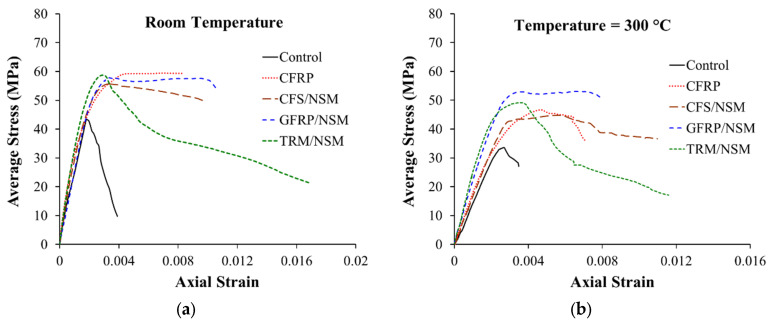
Stress–strain plots for strengthened columns of Ref. [9]: (**a**) at ambient temperature; and (**b**) after elevated temperature exposure of 300 °C.

**Figure 17:**
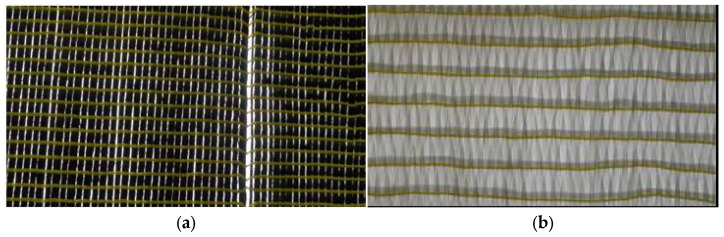
Fabrics utilized in Ref. [9]: (**a**) voids in carbon fibers; and (**b**) no voids in E-glass fibers.

Al-Salloum et al. [9] investigated different strengthening techniques to enhance the strength of RC circular columns at ambient and elevated temperatures. In this investigation, 50 RC columns were prepared and divided into five groups, as shown in Figure 14. The first three groups had 14 columns each, whereas the fourth and fifth groups had 4 columns each. All columns of the first group (control) were left unstrengthened, whereas the columns of the second group (CFRP group) were wrapped using a single continuous layer of CFRP sheet. The columns of the third group (CFS/NSM group) were strengthened using a combination of CFRP strips and NSM steel bars. The four specimens of the fourth group (GFRP/NSM group) were strengthened with a single continuous layer of GFRP in combination with NSM steel rebars. Finally, group five (FRCM/NSM) specimens were wrapped using a combination of continuous FRCM and NSM steel rebars. It should be noted that all strengthening systems were designed to provide roughly the same strength under ambient conditions. For exposure to elevated temperatures, columns were heated in an oven at temperatures ranging from 100 to 800 °C for a 3 h duration (see Figure 15). Subsequent to cooling, the columns were tested in compression. While the design of the strengthening systems ensured comparable strengths at ambient conditions, variations were observed in volumetric strain energy. As shown in Figure 16a, the FRCM/NSM group of columns was most effective in terms of energy absorption and ductility. At ambient temperatures, in order of decreasing ductility, the strengthening systems could be ranked as follows: FRCM/NSM, GFRP/NSM, CFS/NSM, and CFRP. Figure 16b illustrates the stress–strain plots for RC columns after exposure to 300 °C. A comparison of the performance of the strengthening systems for columns at this temperature revealed that the GFRP/NSM system was the best and the next one in terms of strength was the FRCM/NSM system. In an order of decreasing efficiency, the strengthening systems can be arranged as GFRP/NSM, FRCM/NSM, CFRP, and CFS/NSM. Both CFRP-based systems were inefficient when exposed to heating because of the presence of voids within the glass and carbon fabrics (Figure 17). The glass fabric had a custom weave with no voids, whereas the carbon fabric had visibly larger voids. It was concluded in the study that, when heated, the voids transmitted heat to the epoxy matrix, thereby reducing the bond efficiency and resulting in strength deterioration. Conversely, in the GFRP system, the absence of voids between fibers protected the epoxy from heat damage. A similar conclusion was drawn for the FRCM/NSM system because of the protection provided by a layer of mortar.

**Figure 18:**
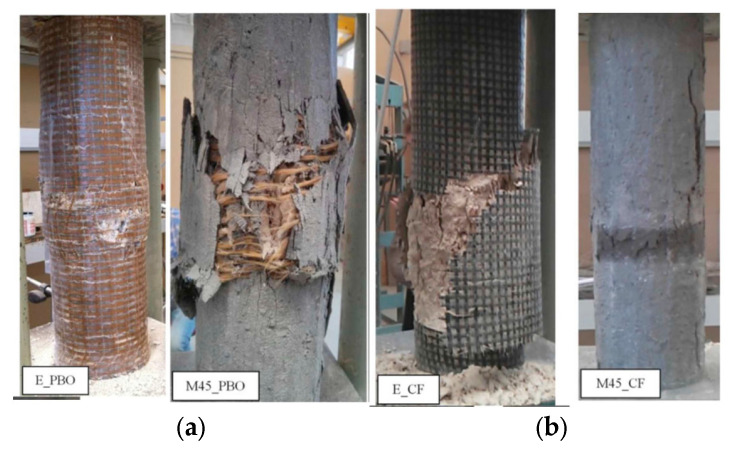
Failure modes for FRP- vs. FRCM-confined cylinders [60]: (**a**) specimens with PBO fabric; and (**b**) specimens with carbon fabric.

**Figure 19:**
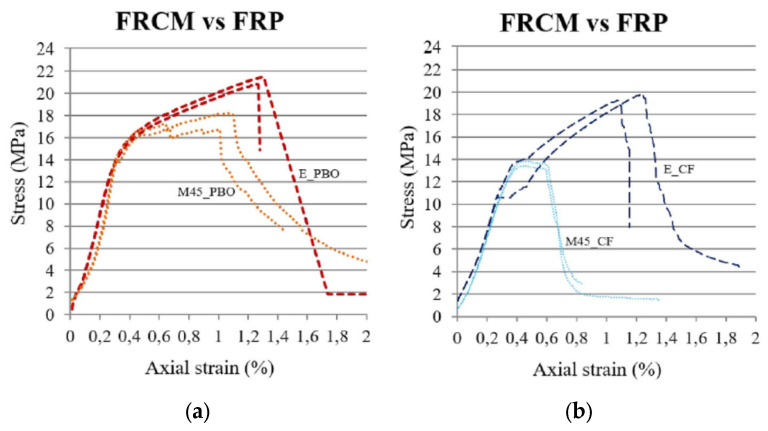
Stress–strain behavior of FRP- vs. FRCM-confined cylinders [60]: (**a**) specimens with PBO fabric; and (**b**) specimens with carbon fabric.

In another study, Donnini et al. [60] conducted experiments to compare three different confinement techniques for concrete members, in which they tested 20 concrete cylinders (140 × 460 mm) under compression. Three strengthening systems employed in this study were based on FRPs, FRCM, and high-performance mortar (HPM). Parameters considered in the study were fabric materials (carbon and PBO) and matrix materials (organic and inorganic). The application of the HPM system as an external jacket of 30 mm thickness was also investigated. The study’s outcome revealed that FRCM was less efficient than FRP, which could be attributed to the weak penetration of the inorganic matrix within the fabric filaments, causing less adhesion of the matrix with fibers. The failure modes for FRP- and FRCM-confined cylinders are depicted in Figure 18. In general, the failure in FRP-wrapped specimens was more explosive and abrupt compared with the failure mode of FRCM-wrapped specimens, wherein a more ductile behavior was observed with the formation of multiple cracks and gradual slip between the fabric and mortar bond. The stress–strain curves of the FRP- and FRCM-retrofitted specimens are shown in Figure 19. As seen from the curves, the behavior of the specimens is diverse except for the curves’ initial part. In the FRP system (carbon and PBO), there was no slip of the fabric, as the epoxy resin prevents it when the confinement is effective and the curve behaves linearly until failure. However, in the cylinders wrapped using the FRCM system, the mortar starts to crack, and slippage of the fabric starts due to the progressive loss of the bond, thereby changing the curve’s gradient. The FRCM effectiveness in this stage is dependent solely on the friction between the fabric and mortar. However, in the case of the PBO fabric, its overall performance is improved as a result of its molecular structure being able to establish bonds with the inorganic mortar. With an inorganic mortar, carbon fibers never develop their full effectiveness owing to their smooth surface, and the absence of chemical bonds between the fiber and mortar, as well as the microscopic behavior of dry carbon fabric. This phenomenon results in a stress–strain behavior for the carbon FRCM system where, once the reinforcement is activated, the stress is approximately steady, and a stress plateau is observed until failure. In contrast, for the PBO-FRCM system, a strain hardening response was noted once the fabric started slipping. The reduction in confined strength in the case of the inorganic matrix in comparison with the organic matrix ranged from 17% for PBO fabrics to 30% for dry carbon fabric.

**Figure 20:**
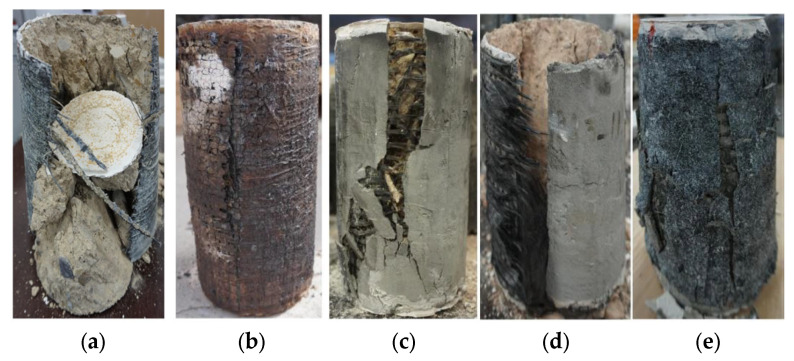
Examples of failure modes of FRP- and FRCM-confined cylinders tested at elevated temperature [61]: (**a**) rupture of FRP jacket at ambient temperature; (**b**) adhesive failure of FRP jacket at elevated temperature; (**c**) rupture of FRCM jacket at ambient temperature; (**d**) rupture of FRCM jacket at elevated temperature; and (**e**) mixed failure of FRCM jacket at elevated temperature.

**Figure 21:**
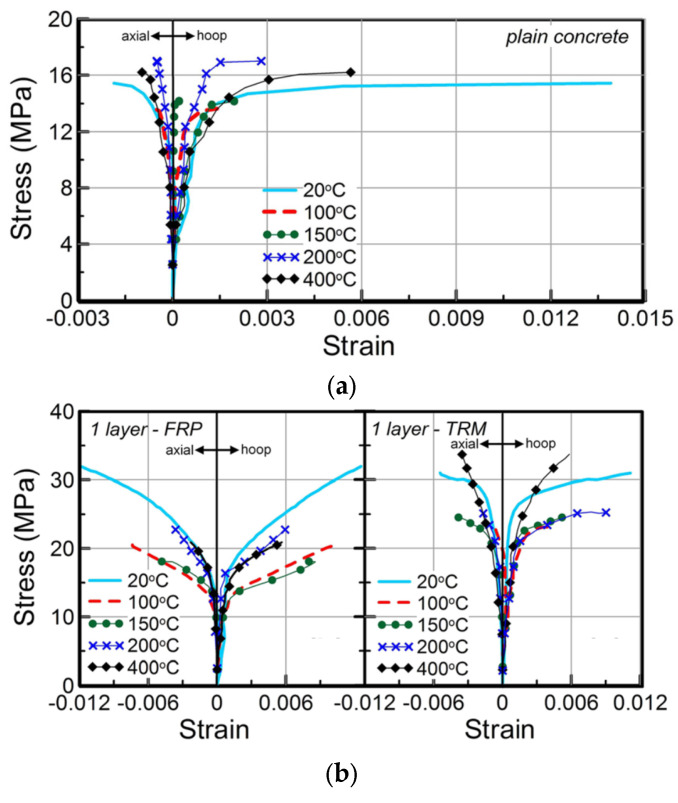

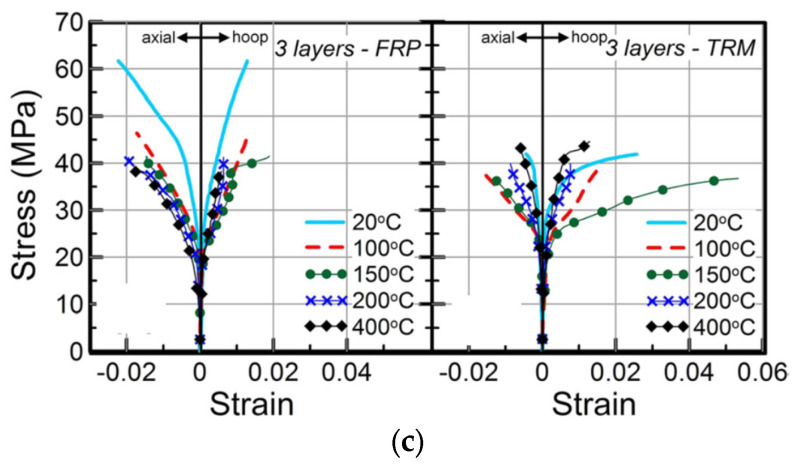
Peak compressive strength vs. temperature for cylinders tested at elevated temperature [61]: (**a**) plain concrete cylinders; (**b**) cylinders having one layer of FRP (or FRCM) composites; and (**c**) cylinders with 3 layers of FRP (or FRCM) composites.

In a series of tests at higher elevated temperatures on 24 concrete cylinders, Cerniauskas et al. [61] studied the efficacy of FRP and FRCM retrofitting in providing confinement to concrete circular columns. The uniaxial compression behavior of different strengthening systems was studied after heating the specimens to temperatures ranging from 20 to 400 °C. The specimens were tested at elevated temperatures. The investigated parameters included the number of textile layers, cementitious mortar vs. epoxy-resin matrix, and elevated temperature (up to 400 °C). A summary of failure modes for all cylinders is depicted in Figure 20. The figure shows that, at ambient temperatures, samples with one and three layers of FRP failed due to the rupture of the FRP jacket, and a similar mode of failure was noted for all FRP-strengthened specimens for temperatures up to 100 °C. The FRP-confined cylinders tested at 150 °C (Figure 20b) showed a mixed mode of failure involving both fiber fracture and bond failure, whereas the failure mode of the cylinders tested at higher temperatures involved bond failure alone because of the decomposition or softening of the epoxy resin, as seen in Figure 20b. Figure 20c shows the failure modes for FRCM-strengthened specimens at ambient temperatures, where the failure was due to the rupture of the FRCM jacket. At temperatures above 100 °C, it was observed that the FRCM-strengthened specimen recovered its strength better than both the unstrengthened and FRP-strengthened specimens. At 400 °C, the specimen jacketed with a single layer of FRCM performed even better than its counterpart tested at ambient temperature. In most cases, as shown in Figure 20d, a single-layer-wrapped FRCM specimen failed as a result of jacket rupture due to excessive hoop stresses. In contrast, for some FRCM cylinders, failure could be a combined effect of jacket rupture and bond failure (see Figure 20e). The stress–strain behavior for all cylinders is summarized in Figure 21, and, as seen from the figure, it was concluded that the strength of FRP-confined cylinders decreased with a rise in temperature; however, the FRP confinement was still effective in providing confinement at all exposure temperatures (even at 400 °C which is well above *T_g_*) in the case of both the single- and three-layered specimens. This performance was attributed to the improved bond resulting from the winding friction, which is relatively more effective for three layers of FRP compared with the single layer. The results from the study concluded that a small loss in the effectiveness of the FRCM strengthening system was observed between 100 and 200 °C, which was attributed to the reduction in concrete’s compressive strength at those temperatures (also verified by the test results of the unconfined concrete after exposure to elevated temperatures). Finally, it was also concluded that both single- and three-layer FRCM strengthening demonstrated improved strength at 400 °C. The study made a vital observation; at elevated temperatures, the number of layers of FRP and FRCM or the lap length has a substantial influence on the efficiency of confinement, and proposed further investigations to study the effect of using more than one continuous wrap on the behavior of FRP- as well as FRCM-confined concrete under elevated temperatures.

### 3.2. Discussion of Main Findings

#### 3.2.1. Overall Behavior

**Figure 22:**
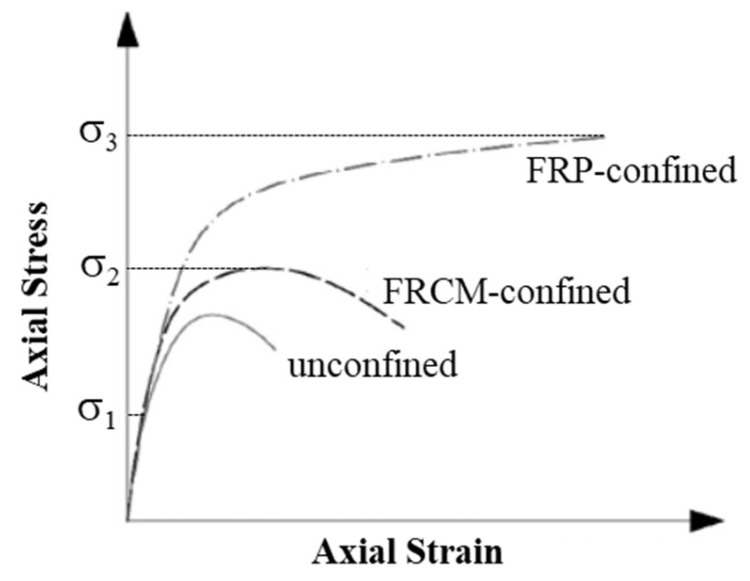
Sketch of stress–strain response of FRP- vs. FRCM-confined concrete [63].

From extensive research performed on FRP and FRCM confined concrete columns, it can be concluded that the tensile properties of the strengthening material have a vital role in the overall behavior of the confined column. This is attributed to the tensile stress generation in the jacket, owing to the increase in the diameter of the column. Figure 22 depicts the main distinctions between columns confined by FRP and FRCM in a quantitative manner, wherein the stress–strain response of unconfined, FRP-confined, and FRCM-confined columns is illustrated (see also [58,62]). Three significant points of stress-values have been identified in the figure and classified as $\sigma_{1}$, $\sigma_{2}$, and $\sigma_{3}$. The unconfined column compressive strength is termed as $\sigma_{1}$, and, when the stress in columns does not exceed $\sigma_{1}$, the column remains intact. The stress at which damage to the column concrete, as well as the FRCM system, is observed is termed as $\sigma_{2}$. At this stage, the inorganic matrix cracks, and the properties of the column are affected by the properties of the inorganic matrix. As the mortar becomes weak, it undergoes premature damage, which leads to the strain softening in the FRCM-confined specimen, though the strains are still higher compared to the unconfined specimen. Once the matrix cracks, the interfacial bond of the fibers with the matrix plays an important role in the stress–strain behavior. As long as the bond is perfect, stresses are progressively transmitted to the fibers, with cracks evolving to cause the complete damage of the mortar. However, there is a sudden stress-transfer to the fibers when the bond level is very low, thereby subjecting them to non-uniform tension, causing early failure. The load for this type of failure is significantly less than the fibers’ tensile strength. The FRP-confined specimen remains undamaged and shows an ascending behavior in the curve.

The stress value corresponding to the FRP-failure (ultimate stress of FRP-confined concrete) is termed as $\sigma_{3}$. At this stage, the FRP-confined column undergoes sudden failure owing to the fracture of FRP fibers and induced debonding. When using FRP confinement, a hardening behavior is expected after the peak stress, continuing until the FRP ruptures. As seen from Figure 22, the FRP system is more efficient compared to the FRCM in terms of both the deformability and the strength. This observation highlights the critical role of the adopted matrix and the quality of interfacial bond. In the case of FRCM, the failure of the mortar and the resulting debonding between the mortar and fibers lead to a reduction in confinement pressure and, consequently, in ultimate strength. In contrast, for FRP, the superior functioning of the epoxy-resin matrix and a perfect bond between the fibers and matrix allows the composite to remain intact until failure, thereby increasing the compressive strength considerably.

#### 3.2.2. FRP vs. FRCM Confinement for Columns Tested at Ambient Temperature

The results of the FRCM-enhanced columns, alongside their FRP equivalents, evaluated by the researchers mentioned previously at ambient temperatures, are included in Table 4. The chart indicates that the FRP-upgraded columns failed owing to concrete crushing as a result of the abrupt fracture of the FRP sheets. Nevertheless, the collapse of the FRCM-strengthened columns was less explosive than the case when the resin-impregnation was used, which is attributable to either the gradual rupture of individual fibers or bond failure of the fiber–matrix interface. To compare FRCM with its FRP counterpart, the effectiveness ratio of FRCM to FRP was computed for all counterparts presented in Table 4 from the following:

(1) $$FRCM\;vs.\;FRP\;effectiveness\;ratio=\frac{f_{cc\;}^{\prime }(for\;FRCM\;upgraded\;column)-f_{co}^{\prime }}{f_{cc}^{\prime }(for\;FRP\;upgraded\;column)-f_{co}^{\prime }}$$

where $f_{co}^{\prime }$ and $f_{cc}^{\prime }$ are the compressive strengths of unconfined and FRCM (or FRP) confined concrete. It should be noted that the column specimens listed in Table 4 were selected such that both the FRP and FRCM systems, which have been compared, have almost the same volumetric ratio.

From the results depicted in Table 4, it is evident that FRP is relatively more effective in increasing the axial capacity of the confined concrete in comparison with FRCM. The effectiveness ratio of FRCM jacketing ranged from 0.2 to 1.38, which increased as the number of FRCM layers was incremented. For the same textile material, the columns upgraded using one or two FRCM layers showed an average effectiveness ratio of 0.62, whereas, for the columns upgraded using three or more FRCM layers, the FRCM vs. FRP effectiveness ratio was 0.86. It is thus suggested to employ at least three FRCM layers for the axial strengthening of concrete columns using cementitious-based composites; however, for FRP composites, one or two layers could be used.

#### 3.2.3. FRP vs. FRCM Confinement for Columns Exposed to High Temperature

For columns exposed to high temperature, the performance of FRCM and FRP strengthening is compared by estimating the FRCM vs. FRP effectiveness ratio using Equation (1). Additionally, both FRCM and FRP effectiveness ratios for elevated temperature exposure are estimated from the following equation:

(2) $$FRCM\;\left(or\;FRP\right)\;effectiveness\;ratio\;due\;to\;heating=\;\frac{f_{cc,T}^{\prime }}{f_{cc,R}^{\prime }}$$

where $f_{cc,R}^{\prime }$ and $f_{cc,T}^{\prime }$ are the compressive strengths of confined concrete at ambient temperature and after elevated temperature exposure. As there are two types of high-temperature exposure tests—(i) those assessing the residual capacity after cooling, and (ii) those conducted at elevated temperature—the results are discussed separately below.

(a) Columns tested for residual capacity after cooling

For the circular columns tested in Ref. [9] for the residual capacity after exposure to 300 °C, the FRCM vs. FRP effectiveness ratio, estimated using Equation (1), is reported in Table 5. Additionally, the FRCM and FRP effectiveness ratios for elevated temperature exposure, calculated using Equation (2), are also summarized in the table. The temperature values reported in the table refer to oven temperatures.

It is noted that both FRCM and FRP exhibited comparable performance as strengthening materials in enhancing the axial capacity of columns exposed to elevated temperature environments. The FRCM-strengthened columns exposed to high temperature maintained an effectiveness ratio of 84%, compared to their effectiveness ratio at room temperature. The performance of FRP-strengthened concrete subjected to high temperature was also comparable to the FRCM-upgraded columns. As depicted in Table 5, FRP-strengthened specimens have an effectiveness ratio ranging from 80% to 92% (with an average value of 84%). Despite heating, the peak strength of FRP-upgraded specimens exposed to a high temperature of 300 °C was significantly more than the strength of the unconfined specimens. This finding was also supported by previous research on the residual capacity of FRP-strengthened concrete columns when exposed to heating [64,65] in which externally bonded FRP composites could enhance the load-carrying capacity of concrete columns in cases where that the temperature at the FRP level is not more than the decomposition temperature of the epoxy resin (345 °C for the epoxy used in the study).

(b) Columns tested at elevated temperature

For the circular columns tested by Cerniauskas et al. [61] under a steady-state thermal regime between 100 and 400 °C, the FRCM vs. FRP effectiveness ratio, estimated using Equation (1), is summarized in Table 6. Moreover, the FRCM and FRP effectiveness ratios for elevated temperature exposure, calculated using Equation (2), are also given in the table (the temperatures again correspond to the oven readings).

It is noted that FRCM exhibited good performance as a strengthening material in enhancing the axial capacity of columns exposed to elevated temperature environments. In fact, the FRCM-strengthened columns exposed to high temperature maintained an effectiveness ratio ranging from 75% to 107% (with an average value of 88%), compared to their effectiveness ratio at room temperature. However, the performance of FRP-strengthened concrete subjected to high temperature was worse than that of FRCM-upgraded columns. As depicted in Table 6, FRP-strengthened specimens have an effectiveness ratio ranging from 56% to 75% (with an average value of 66%). Moreover, it was found that the peak strength of FRP-upgraded specimens exposed to high temperatures of 100 °C to 400 °C was significantly more than the strength of unconfined specimens. This observation is consistent with the earlier findings [64,65] that FRP confinement remains beneficial up to temperatures approaching the epoxy decomposition threshold (400 °C for the epoxy used in the study).

When the exposure temperature exceeds the glass transition temperature (*T_g_*) of the epoxy in FRP systems, the strength of the FRP, and, hence, its confinement effectiveness, reduces similarly whether tested at an elevated temperature or after cooling. However, because the residual strength of concrete itself decreases after cooling [66], the overall strength of FRP-confined concrete tested after cooling is expected to be lower. For FRCM systems, although they contain no epoxy, their matrix and fiber properties also deteriorate at high temperatures, leading to a reduced confinement capacity. Furthermore, since the residual concrete strength after cooling is lower, the strength of the FRCM-confined concrete tested after cooling is also expected to be less than that tested at an elevated temperature. Therefore, the strengths of the confined concrete tested after cooling and those tested at an elevated temperature cannot be compared, and further studies are recommended to quantify these effects. If future investigations confirm that testing after cooling yields conservative results for FRP- and FRCM-confined concrete compared to specimens tested at elevated temperatures, it would, as in the case of unconfined concrete [66], eliminate the need for testing at elevated temperatures and, thus, save significant experimental efforts and resources.

## 4. Prediction Models for FRCM-Confined Concrete Columns

The nature of the FRCM matrix (organic or inorganic) is different from that of the FRP matrix, thereby creating different interface (matrix and fabric) behaviors, failure modes, and micro-mechanical effects that are dissimilar to those of the FRP matrix. As a result, even though numerous models for predicting the stress–strain behavior of FRP-confined concrete in compression exist in the literature (e.g., [63,67,68,69,70,71,72,73,74,75,76,77,78,79,80,81,82,83,84,85,86,87,88,89,90,91,92,93,94,95,96,97,98]), those models cannot be employed to describe the stress–strain behavior of FRCM-confined concrete. One major dissimilarity between the FRCM and FRP matrix is that the resin in the FRP composite is immune to cracking, thereby avoiding the slipping of fibers from the matrix; however, for the FRCM systems, the cementitious matrix which has a low tensile strength does crack even at low levels of loading, and failure is thereby often governed by the slipping of the fabric from the matrix. As a result, models proposed for FRP systems do not predict the FRCM composite behavior with any degree of accuracy or reliability, and are therefore not applicable for predicting the behavior of FRCM composites under compression loading. New analytical models are proposed by some researchers [6,14,59,63,99,100], and Ombres and Mazzuca [100] considered the different behavior of inorganic-based systems. All of these models are meant for circular columns; however, the models of Triantafillou et al. [14] and ACI 549.4R-13 [6] can also assess the stress–strain response of rectangular FRCM-confined concrete. Table 7 lists the equations proposed by the six FRCM-confined models for the prediction of the confined compressive strength ($f_{cc}^{\prime }$) and ultimate compressive strain ($\varepsilon_{ccu}$) of FRCM-confined cylindrical concrete columns. In these formulas, $f_{lu}$ is the lateral confining pressure given by the following:

(3) $$f_{lu}=0.5\rho_{f}E_{f}\varepsilon_{fe}$$

where $\rho_{f}=4n_{f}t_{f}/D$ (with $n_{f}$ = number of fabric layers, $t_{f}$ = equivalent smeared thickness of fabric layer, and *D* = column diameter); $E_{f}$ = tensile elastic modulus of fabric material; and $\varepsilon_{fe}$ = effective fabric strain, given by

(4) $$\varepsilon_{fe}=k_{e}\varepsilon_{fu}\leq 0.012$$

where $\varepsilon_{fu}$ = strain at rupture of fabric reinforcement; and $k_{e}$ = strain efficiency factor, calculated as proposed by Ombres and Mazzuca [100] from the following formula:

(5) $$k_{e}=0.25\left[{\left(\frac{\rho_{f}E_{f}}{f_{co}^{\prime }}\right)}^{0.3}-1\right]$$

Moreover, in the equations used for calculating the ultimate compressive strain, the parameter $\varepsilon_{co}$ is the compressive strain of the unconfined concrete corresponding to $f_{co}^{\prime }$ (can be taken as 0.002).

## 5. Conclusions and Recommendations

This section presents the main conclusions of this state-of-the-art review article with respect to the use of FRP versus FRCM composites in the confinement of RC columns. In addition, this section outlines suggestions for future research to cover the gaps in the current knowledge. The conclusions derived from this review report are summarized in the following:

1. A considerable gain in axial compressive strength and ductility was observed for specimens confined using FRCM jackets and tested under uniaxial compression. The gain in strength was proportional to the number of layers, and was dependent on the tensile strength of mortar, which determined the ultimate mode of failure to be either fiber rupture or debonding.
2. As discussed within the review report, the effectiveness of FRCM jackets was to a much lesser extent when compared with their resin-impregnated counterpart, FRP, by an order of approximately 74% for strength and 83% for ultimate strains.
3. Columns upgraded with one or two FRCM layers displayed the least FRCM versus FRP effectiveness ratio, with an average ratio of 0.62. However, for the columns strengthened with three or more FRCM layers, the average effectiveness ratio was 0.86.
4. The failure modes of FRCM-confined specimens were far less abrupt and less explosive compared to those of FRP-confined specimens. This could be attributed to the rupture of individual fiber bundles, which is of a slow progressing nature.
5. Compared to its effectiveness at ambient temperatures, FRCM composites display good performance as a confining material even at elevated temperatures, wherein the axial capacity of concrete columns was increased, and an effectiveness ratio of 84% was maintained after exposure to a temperature of 300 °C for 3 h.
6. FRP-strengthened columns performed well after their exposure to 300 °C for three hours, as they maintained from 80% to 92% of their axial capacity at room temperature. In fact, externally bonded FRP composites were capable of increasing the axial load capacity of RC columns on the condition that the epoxy resin decomposition temperature is not exceeded at the FRP level.
7. In view of the design of strengthening schemes for the axial strengthening of RC columns, FRCM composites may be used. However, when designing the FRCM systems, it is recommended to use the properties of the bare textile alone, ignoring the contribution of the mortar. When it comes to the number of layers, at least three FRCM layers for the confinement of RC columns, along with polymer-modified cementitious mortar, should be used. In this case, the analytical models available in the literature (summarized in Table 7) can be directly applied to design an FRCM composite system for the axial strengthening of circular RC columns. However, reliable confinement models are not available for FRCM-strengthened square and rectangular columns. In this regard, the design guidelines used for the axial strengthening of RC columns using externally bonded FRP composites may be directly applied to FRCM composites, but with the introduction of a strength knockdown factor of 0.64 (estimated from Table 4 as *m* − *σ*, where *m* = mean value and *σ* = standard deviation).

After an extensive review of the accessible literature on the effectiveness of FRCM composites in strengthening RC columns in comparison with FRP, it was observed that further research is required to fill the gaps that exist in the current knowledge. We therefore have the following recommendations:

1. The behavior and performance of FRCM-strengthened RC columns need to be studied during exposure to the standard fire test as per ASTM E119 [101].
2. We must conduct more experimental work on FRCM-strengthened square and rectangular columns, and, hence, devise analytical confinement models.
3. The efficiency of FRCM compared with externally bonded FRP composites for strengthening wall-like RC columns under axial compression needs to be experimentally studied.
4. Future research should focus on developing standardized thermal exposure protocols, including defined temperature ramp rates, hold durations, and the distinction between oven setpoints and in situ temperatures at the jacket or core.
5. Further studies are also needed to examine the confinement behavior of rectangular sections with varying corner radii and anchorage configurations to optimize the stress transfer and prevent premature debonding.
6. The long-term durability of FRCM systems should be evaluated under cyclic wet–dry and freeze–thaw conditions for different mortar compositions to better understand the degradation mechanisms and ensure a reliable performance in aggressive environments.
7. Further experimental studies are recommended to enable a reliable comparison of the confinement effectiveness of FRP- and FRCM-confined concrete tested at elevated temperatures and after cooling.

## Figures and Tables

**Table 1:** Performance comparison between aramid, glass, and carbon fibers [31].

Criterion	Performance Level of Different Types of Fibers *		
	E-glass	Aramid	Carbon
Compressive strength	G	I	VG
Tensile strength	VG	VG	VG
Long-term response	A	G	VG
Modulus of elasticity	A	G	VG
Fatigue performance	A	G	VG
Resistance to alkaline environment	I	G	VG
Unit weight	A	E	G
Cost	VG	A	A

* A: adequate, E: excellent, G: good, I: inadequate, VG: very good.

**Table 2:** Mechanical properties of some commercially available mortars [49].

Mortar Type	Trade Name	Supplier	28-Day Compressive Strength (MPa)		28-Day Tensile Strength (MPa)
			Lab	Data Sheet	Lab
Polymer-modified cementitious mortar	Renderoc S	Fosam	62.3	55.0	6.90
	SikaRep	Sika	56.4	45.0	3.40
	Mapegrout Thixotropic	Mapei	66.0	60.0	3.94

**Table 3:** Summary of the properties of different FRCM systems tested in literature [55].

Textile Used	E_I_ (GPa)	E_II_ (GPa)	E_III_ (GPa)	Ultimate Tensile Strength, f_t_ (MPa)	Ultimate Tensile Strain, ε_t_ (%)
Carbon	460–1575	68	50–186	970–1500	0.74–1.8
Glass	260–1310	16–71	55–110	870–1980	0.69–1.92
PBO	1180–1805	76	120–220	1550–3320	1.4–1.8

**Table 4:** Comparison between FRCM- and FRP-strengthened columns in axial compression and tested at ambient temperature *.

Reference	Shape of Column	Control Column		FRCM-Strengthened Column					FRP-Strengthened Column					FRCM/FRP	
		Col ID	$f_{co}^{\prime }$(MPa)	Col ID	$n_{f}$	$f_{cc}^{\prime }$(MPa)	$\varepsilon_{ccu}$	Failure Mode	Col ID	$n_{f}$	$f_{cc}^{\prime }$(MPa)	$\varepsilon_{ccu}$	Failure Mode	Effectiveness Ratio	Ultimate Strain Ratio
[56]	C	-	54.0	-	2	100	0.0295	RP	-	2	105	0.0394	RP	0.90	0.75
[36]	C	B_C	21.8	B_MII2	2	27.4	0.0098	RP	B_R2	2	33.5	0.0167	RP	0.48	0.59
	C	B_C	21.8	B_MII3	3	32.4	0.0108	RP	B_R3	3	41.9	0.0255	RP	0.53	0.42
	S	C_C	14.3	C_MII2	2	20.0	0.0118	RP	C_R2	2	18.4	0.0124	RP	1.38	0.95
	S	C_C	14.3	C_MII4	4	21.6	0.0176	RP	C_R4	4	21.0	0.0203	RP	1.09	0.87
[57]	S	U_C	15.3	U_M4	4	26.6	0.0076	RP	U_R2	2	30.6	0.0082	RP	0.74	0.93
	S	U_C	15.3	U_M6	6	31.6	0.0106	RP	U_R3	3	34.7	0.0128	RP	0.84	0.83
	S	s200_C	22.9	s200_M4	4	34.2	0.0112	RP	s200_R2	2	37.3	0.0128	RP	0.79	0.88
	S	s200_C	22.9	s200_M6	6	36.0	0.0133	RP	s200_R3	3	44.7	0.0148	RP	0.60	0.90
	S	s100_C	24.1	s100_M4	4	38.3	0.0126	RP	s100_R2	2	42.0	0.0132	RP	0.79	0.95
	S	s100_C	24.1	s100_M6	6	39.9	0.0150	RP	s100_R3	3	45.2	0.0172	RP	0.75	0.87
[59]	C	Control	20.4	LDG-A	2	26.9	0.0090	DB	LDG-E	2	36.7	0.0228	RP	0.40	0.39
	C	Control	20.4	LDG-H	2	30.0	0.0079	DB	LDG-E	2	36.7	0.0228	RP	0.59	0.35
	C	Control	20.4	HDG-A	2	24.5	0.0135	DB	HDG-E	2	40.8	0.0157	RP	0.20	0.86
	C	Control	20.4	HDG-H	2	30.0	0.0075	DB	HDG-E	2	40.8	0.0157	RP	0.47	0.48
	C	Control	20.4	BGP-A	2	28.8	0.0027	DB	BGP-E	2	33.5	0.0177	RP	0.64	0.15
	C	Control	20.4	BGP-H	2	31.8	0.0068	DB	BGP-E	2	33.5	0.0177	RP	0.87	0.38
[9]	C	C	43.1	FRCM/NSM	3	58.7	0.0168	RP	CF	1	58.0	0.0076	RP	1.05	2.21
	C	C	43.1	FRCM/NSM	3	58.7	0.0168	RP	CFS/NSM	1	55.7	0.0099	RP	1.24	1.7
	C	C	43.1	FRCM/NSM	3	58.7	0.0168	RP	GF/NSM	1	57.8	0.0106	RP	1.06	1.58
[60]	C	Ref	11.405	M45_PBO	1	17.705	0.010895	RP	E_PBO	1	21.16	0.01265	RP	0.65	0.86
	C	Ref	11.405	M45_CF	1	13.655	0.004845	RP	E_CF	1	19.57	0.01202	RP	0.28	0.40
Average of whole data														0.74 ± 0.30	0.83 ± 0.48
Average for one or two layers of FRCM														0.62 ± 0.33	0.56 ± 0.26
Average for three or more layers of FRCM														0.86 ± 0.22	1.10 ± 0.51

* C = circular column; S = square column; $f_{co}^{\prime }$ = compressive strength of unconfined concrete; $n_{f}$ = no. of FRCM (or FRP) layers; $f_{cc}^{\prime }$ = compressive strength of FRCM (or FRP)-confined concrete; $\varepsilon_{ccu}$ = peak axial strain for FRCM (or FRP)-confined concrete; RP = fiber rupture; DB = debonding at matrix–fiber interface.

**Table 5:** Comparison between FRCM- and FRP-strengthened columns in axial compression and tested for residual capacity after high temperature exposure.

Reference	Exposure Temp. (°C)	$f_{co,T}^{\prime }$(MPa)	FRCM-Strengthened Column					FRP-Strengthened Column					FRCM/FRP		FRCM Effectiveness Due to Heating	FRP Effectiveness Due to Heating
			Col ID	$n_{f}$	$f_{cc,T}^{\prime }$(MPa)	$\varepsilon_{ccu,T}$	Failure Mode	Col ID	$n_{f}$	$f_{cc,T}^{\prime }$(MPa)	$\varepsilon_{ccu,T}$	Failure Mode	Effectiveness Ratio	Ultimate Strain Ratio		
[9]	20	43.1	FRCM/NSM	3	58.7	0.0168	RP	CF	1	58	0.0076	RP	1.05	2.21	1	1
	300	34.4	FRCM/NSM-300	3	49.2	0.0116	Mixed	CF-300	1	46.7	0.005	Mixed	1.20	2.32	0.84	0.81
	300	34.4	FRCM/NSM-300	3	49.2	0.0116	Mixed	CFS/NSM-300	1	44.8	0.0068	Mixed	1.42	1.71	0.84	0.80
	300	34.4	FRCM/NSM-300	3	49.2	0.0116	Mixed	GF/NSM-300	1	53.1	0.008	Mixed	0.79	1.45	0.84	0.92
Average of data tested after high temperature exposure															0.84 ± 0.00	0.84 ± 0.07

**Table 6:** Comparison between FRCM- and FRP-strengthened columns in axial compression and tested at high temperature exposure.

Reference	Exposure Temp. (°C)	$f_{co,T}^{\prime }$(MPa)	FRCM-Strengthened Column					FRP-Strengthened Column					FRCM/FRP		FRCM Effectiveness Due to Heating	FRP Effectiveness Due to Heating
			Col ID	$n_{f}$	$f_{cc,T}^{\prime }$(MPa)	$\varepsilon_{ccu,T}$	Failure Mode	Col ID	$n_{f}$	$f_{cc,T}^{\prime }$(MPa)	$\varepsilon_{ccu,T}$	Failure Mode	Effectiveness Ratio	Ultimate Strain Ratio		
[61]	20	16.1	-	1	31	0.0055	RP	-	1	32.5	0.012	RP	0.91	0.46	1.00	1.00
	100	13.6	-	1	23.2	0.00074	RP	-	1	20.5	0.0074	Mixed	1.39	0.10	0.75	0.63
	150	14.2	-	1	24.5	0.004	Mixed	-	1	18.2	0.0048	Mixed	2.58	0.83	0.79	0.56
	200	17.1	-	1	25.6	0.00177	RP	-	1	22.9	0.00352	Adhesive	1.47	0.50	0.83	0.70
	400	16.2	-	1	31.5	0.0035	Mixed/RP	-	1	21.1	0.0023	Adhesive	3.12	1.52	1.02	0.65
	20	16.1	-	3	42.1	0.004	RP	-	3	62.8	0.0221	RP	0.56	0.18	1.00	1.00
	100	13.6	-	3	38.6	0.0152	Mixed	-	3	46.8	0.0168	RP	0.75	0.90	0.92	0.75
	150	14.2	-	3	36.9	0.0121	RP	-	3	41.9	0.014	Mixed	0.82	0.86	0.88	0.67
	200	17.1	-	3	40.5	0.008	RP	-	3	41.8	0.019	Adhesive	0.95	0.42	0.96	0.67
	400	16.2	-	3	45	0.0053	Mixed	-	3	38.5	0.0172	Adhesive	1.29	0.31	1.07	0.61
Average of data tested after high temperature exposure															0.90 ± 0.11	0.66 ± 0.06

**Table 7:** Analytical models for FRCM-confined circular concrete columns.

Reference	$\mathrm{Formula}\;\mathrm{for}\;f_{cc\;}^{\prime }$	$\mathrm{Formula}\;\mathrm{for}\;\varepsilon_{ccu}$
[14]	$f_{cc}^{\prime }=f_{co}^{\prime }\left(1+1.9{\left(\frac{f_{lu}}{f_{co}^{\prime }}\right)}^{1.27}\right)$	$\varepsilon_{ccu}=\varepsilon_{co}\left(1+\frac{0.046}{\varepsilon_{co}}{\left(\frac{f_{lu}}{f_{co}^{\prime }}\right)}^{1.44}\right)$
[59]	$f_{cc}^{\prime }=f_{co}^{\prime }\left(1+2.87{\left(\frac{f_{lu}}{f_{co}^{\prime }}\right)}^{0.775}\right)$	$\varepsilon_{ccu}=\varepsilon_{co}\left(1+\frac{0.046}{\varepsilon_{co}}{\left(\frac{f_{lu}}{f_{co}^{\prime }}\right)}^{0.775}\right)$
[6]	$f_{cc}^{\prime }=f_{co}^{\prime }\left(1+3.1\frac{f_{lu}}{f_{co}^{\prime }}\right)$	$\varepsilon_{ccu}=\varepsilon_{co}\left(1+12\left(\frac{f_{lu}}{f_{co}^{\prime }}\right){\left(\frac{\varepsilon_{fe}}{\varepsilon_{co}}\right)}^{0.45}\right)$
[99]	$f_{cc}^{\prime }=f_{co}^{\prime }\left(1+5.286\frac{f_{lu}}{f_{co}^{\prime }}\right)$	$\varepsilon_{ccu}=\varepsilon_{co}\left(\frac{0.041}{\varepsilon_{co}}{\left(\frac{f_{lu}}{f_{co}^{\prime }}\right)}^{0.25}-1.02\right)$
[63]	$f_{cc}^{\prime }=f_{co}^{\prime }+6.7f_{lu}^{0.587}$	$\varepsilon_{ccu}=\varepsilon_{co}\left(1+{\left(\frac{f_{lu}}{f_{co}^{\prime }}\right)}^{0.5}\right)$
[100]	$f_{cc}^{\prime }=f_{co}^{\prime }\left(1+0.913{\left(\frac{f_{lu}}{f_{co}^{\prime }}\right)}^{0.5}\right)$	$\varepsilon_{ccu}=\varepsilon_{co}\left(1+0.963\left(\frac{f_{lu}}{f_{co}^{\prime }}\right){\left(\frac{\varepsilon_{fe}}{\varepsilon_{co}}\right)}^{0.5}\right)$

## Data Availability

All data and models generated or used during the study appear in the article.
